# The changing shape of vaccination: improving immune responses through geometrical variations of a microdevice for immunization

**DOI:** 10.1038/srep27217

**Published:** 2016-06-02

**Authors:** Michael Lawrence Crichton, David Alexander Muller, Alexandra Christina Isobel Depelsenaire, Frances Elizabeth Pearson, Jonathan Wei, Jacob Coffey, Jin Zhang, Germain J. P. Fernando, Mark Anthony Fernance Kendall

**Affiliations:** 1 The University of Queensland, Delivery of Drugs and Genes Group (D2G2), The Australian Institute for Bioengineering and Nanotechnology, St Lucia, QLD 4072, Australia; 2 ARC Centre of Excellence in Convergent Bio-Nano Science and Technology, The University of Queensland, Australia; 3 The University of Queensland, Faculty of Medicine and Biomedical Sciences, Royal Brisbane and Women’s Hospital, Herston, Queensland 4006, Australia

## Abstract

Micro-device use for vaccination has grown in the past decade, with the promise of ease-of-use, painless application, stable solid formulations and greater immune response generation. However, the designs of the highly immunogenic devices (e.g. the gene gun, Nanopatch or laser adjuvantation) require significant energy to enter the skin (30–90 mJ). Within this study, we explore a way to more effectively use energy for skin penetration and vaccination. These modifications change the Nanopatch projections from cylindrical/conical shapes with a density of 20,000 per cm^2^ to flat-shaped protrusions at 8,000 per cm^2^, whilst maintaining the surface area and volume that is placed within the skin. We show that this design results in more efficient surface crack initiations, allowing the energy to be more efficiently be deployed through the projections into the skin, with a significant overall increase in penetration depth (50%). Furthermore, we measured a significant increase in localized skin cell death (>2 fold), and resultant infiltrate of cells (monocytes and neutrophils). Using a commercial seasonal trivalent human influenza vaccine (Fluvax 2014), our new patch design resulted in an immune response equivalent to intramuscular injection with approximately 1000 fold less dose, while also being a practical device conceptually suited to widespread vaccination.

Looking beyond the needle and syringe, novel mechanical means of introducing vaccine into the body have been emerging over the past decade; gene guns^1^,^2^, solid formulations (either aerosol or injected)^3^,^4^ and microneedles/microprojections^5^,^6^,^7^ represent a few of the most promising. These deposit vaccine in close vicinity to much larger densities of immune cells in the skin, compared with the low densities in muscle tissue, targeted by the needle and syringe (a technology that has seen minimal change over the past 160 years). Yet to enter the skin, substantial energy is necessary either due to the size or number of particles/needles/formulations to be deposited. For example, spring applied Nanopatches and helium gas powered gene guns–distinct methods of engaging with the skin–both (interestingly) require about 90 mJ for application^2^,^8^. However the penetration outcome is very different: the Nanopatch projections reach well into the dermis (delivering vaccine to both the epidermis and dermis), whereas the gene gun microparticles lodge primarily into the epidermis^8^,^9^,^10^. Here, we explore how geometry changes can help to change the performance of a microdevice and allow it to generate stronger immune response against a commercial influenza vaccine.

To date, the differences in energy transfer also extend to the cell death profiles that have been shown to correlate with immune responses in the skin^11^. Tissue damage has also been proposed as a mechanism that ensures sporozite uptake by mosquitos, in Malaria^12^. This leads us to question whether energy applied to skin can be better utilized to achieve even further improved immune responses, retaining pain-free approaches. Within the microneedle field, our work and that of others have demonstrated the need and challenges in placing vaccine in tightly defined skin locations^6^, particularly as the question of the ideal immune targeting location in the skin remains in debate^13^. Our work has previously shown that due to the disparity in the mechanical elastic modulus of skin’s constituent layers, entering the skin (or other tissue such as mucosae) in a way that is precise can be challenging^8^,^14^,^15^,^16^.

In this communication, we explore a way to overcome this, with a distinct geometry of micro-device conceived to more effectively transfer energy into skin penetration while concurrently opening up further increases in immunogenicity.

We commenced this study with our existing reference point: the Nanopatch, an array of microprojections 110 μm long (cylindrical, with a conical tip down to 1 μm radius tip), made from silicon with a density of 20,000 per cm^−2^ 17. Whilst this device has given excellent immunogenicity results in mice^6^, its high density necessitates a significant amount of energy to position vaccine in precise dermal and epidermal locations (around 90 mJ for a 4 × 4 mm patch)^8^. With the view of extending this targeting ability, we conceived a novel shape of the projections to be used in the skin, and their density. The aim was to: 1) reduce the number of discrete penetrations that had to be made upon entry to the skin; 2) increase the surface area of protrusion in the skin; and 3) exploit surface crack propagation to enhance penetration and reduce the overall energy required to puncture the skin. The resulting shape of protrusion is shown in Fig. 1a, a tapering extended hexagon shape, with a rapidly tapering tip at the distal end. These protrusions have a spacing of 100 μm between adjacent lines and 80 μm between successive protrusions (giving 8000 cm^−2^). The tip of these protrusions tapered to a distal line ~25 μm long and 1–2 μm wide. This novel design of protrusion on a patch, has been termed the Transdermal Patch (TP). The total number of projections on the Nanopatch (Fig. 1b, NP) was 3364 and on the TP was 1280.

We commenced by coating these and applying them to mouse ear skin, using methods previously described in Chen *et al.*^18^ and Crichton *et al.*^8^ respectively. Figure 1 shows the projections/protrusions before (a, b) and after coating (c, d)–with the coating solution spread over the full surface area of the patch (tips and base). This shows a smooth coating applied to the patches, including around their tips, does not significantly change the geometry of either patch array. Post-application to skin (Fig. 1e,f), it is clear that the coating on the tips of both the NP projections and TP protrusions has been removed, with a clear indication of the extent to which they entered the skin. Both types of patch showed varied penetration over the array surface Figure S1, indicating the complexity of penetrating a topographically variable skin surface. Yet both patches showed significant release from their surface into the skin. We did not observe any projection or protrusion breakage, with the material properties being sufficiently strong to puncture skin at the energy at which they were applied. This is consistent with our studies on the material properties of these devices and skin^19^.

Figure 1g–j shows the top view and cross-section views of delivered vaccine with fluorescent microspheres indicating the location from the two patch designs. The TP (g, h) shows a deeper penetration with delivery spanning the full epidermal and dermal thickness, whereas the NP (i, j) targets the epidermis and upper dermis. Additionally, qualitatively there is a greater overall surface coverage of delivered vaccine by the TP when viewed from above (Fig. 1g,i).

To explore the mechanism of surface puncture, we investigated the *in-situ* effect of projections in the skin using CryoSEM (described previously in Crichton^8^), which involved freezing skin with projections/protrusions in place and then removing or fracturing the patch and skin. Figure 1k shows that clear puncture marks are created in the TP applications (with similar patterns previously shown for NP applications)^20^, where the skin appears to have been entered without large amounts of disruption of the adjacent tissue. For the TP, when the patch was removed immediately after application, the holes quickly started to close (Fig. 1l, within a minute following removal). In observing the skin by CryoSEM following TP application (n = 2 patches for visualization), we infer the strain on the skin surface during application, from the immediate recovery of the tissue after projection removal: 1) In the major axis of the protrusions the length of hole created in the skin is ~36 μm when the projection is *in-situ*, and shrinks to ~25–33 μm after removal, indicating that residual stress decreases the hole size by ~10–30%; 2) In the minor axis, the hole size starts at ~20 μm diameter and reduces to ~5–10 μm, which is a decrease of 50–75%. These relative decreases in size indicate much larger residual stress opening the hole across the major axis, than along it. Further, this supports the hypothesis that crack growth along the major protrusion axis will be the main driver of puncture, which would serve to reduce residual stress concentrations along this axis. In contrast, the Nanopatch projections leave circular holes^8^ that will start to close from all directions, with considerable residual stress from all sides on the projection.

Using *in-situ* freeze-fracturing of skin and patches^20^ we observe the skin condition during TP presence (Fig. 1m). Comparing this to our previous results with the NP^20^ a clear contact edge is visible, which we propose is the main cutting edge that effects the more efficient puncture of the TP.

When we measured Trans-Epidermal Water Loss (TEWL) to confirm the effect of the observed pore closure behavior, Fig. 1n. We observed that the NP had a more rapid return to baseline TEWL than the TP, which we propose is due to the larger cracks that were formed during TP application, causing greater overall TEWL. However, comparing the trends of TEWL for both patches from 2 hours onwards, the rate of TEWL decrease is very similar–reductions of 2.74 g/hm^2^ for the TP and 2.55 g/hm^2^ for the NP. This implies that rate of healing is similar for both patches.

Skin vaccine delivery depth was quantified by measuring the depth to which the fluorescent dye reached within the skin. This showed (Fig. 1o) that NP projections delivered to a depth of 39.9 ± 16. 4 μm (n = 5 mouse ears, 337 measurements) and TP protrusions reached 59.7 ± 20.9 μm (n = 5 mouse ears, 386 measurements). As these distinct penetration measurements have been achieved by using a consistent energy of application (90 mJ), it is clear that the TP protrusions have been considerably more efficient in penetrating the skin (i.e., more depth from the same energy). From these insights on penetration, a greater delivered vaccine dose would be expected on the TP, which was observed in Fig. 1p. We propose that the difference between delivery for the low and high dose are likely to be a result of the different viscosity and/or surface tension that is present with different volumes of vaccine in the coating formulation which has been observed previously^21^. Increasing the total protein content coated on the patch has a dramatic effect on the protein concentrations during the patch coating/dry down. These high protein concentrations affect the viscosity/surface tension during the coating process and rehydration rate when the patch is delivered to the animal. All these factors will affect the delivery efficiencies for high and low vaccine doses.

To confirm that the increased penetration of the TP was due solely to the geometrical design changes of the protrusions and not simply changes to their number or volume in skin, we constructed a 3D model of a single TP protrusion and NP projection in Solid Edge and used it to calculate the model’s surface area (excluding the base) and volume. This surface area was then multiplied by the number of protrusions on the TP or projections on the NP, giving a total surface area in the skin for each patch. Figure S2 shows the surface area within the skin for a single TP or NP, at a particular penetration depth. Using this approach with both surface area and volume, we calculated that for a single projection/protrusion, the volume entering the skin is around 2–3 times greater on the TP protrusions than on the NP. However, due to the larger number of projections on the NP, the overall volume in the skin summed over the whole patch is very similar (within 10%) between the NP and TP, for a particular depth, up to ~50 μm into the skin. This trend is also followed for the surface area of the patches, whereby a similar surface area (within 10%) is placed into the skin by either patch, up to a depth of around 50 μm. From this we can infer that the increased penetration of the TP protrusions into the skin results from a greater ability to penetrate the skin, rather than a disproportionate volume or surface area penetrating into the skin at comparable depths.

To understand how the forces from projections/protrusions impacted the biological activity of the skin when applied, we used cellular death staining and quantified cellular infiltration around the application site. Figure 2 shows the TP and NP cell death profiles when patches were applied to mouse ears. Around the NP projections, we observed dead cells within a radius of ~20–30 μm (n = 4 mouse ears), with a greater number on the edge of the patch where bridged vaccine formulation was restricting penetration but allowing a significant force transmission into skin; these results reflect our previous observations^11^. Around the TP however, dead cells are predominantly present across the major axis of the protrusions. These extend 5–25 μm (n = 5 mouse ears) from the centerline of the protrusions, indicating that this is the area of highest stress during insertion of projections (reflecting Fig. 1l). Furthermore, on the edges of the TP where there is a higher stress (due to the Hertzian contact mechanics)^22^, and where there is some bridging of coating between projections, we observe the fractures of adjacent projections joining to form lines of continuous surface fracture, bordered by necrotic cells. This supports our hypothesis of the stress distribution in the skin and the crack growth that makes it easier for the TP protrusions to enter the skin. Quantifying the level of cellular death around projections/protrusions (Fig. 2b,c), each TP protrusion in the central patch area had 54.7 ± 18.6 dead cells surrounding it, whereas each NP projection had only 21.8 ± 3.0. On the edges of the patches, the TP field of views (420 × 420 μm) had 17.6% more cell death than the NP FOVs. In central patch areas the TP FOVs had 42.7% more cell death than the NP FOVs. These values remain significantly below the levels that were observed during another skin vaccination technique, the gene gun, where a density of 2 microparticles per 1000 μm^2^ resulted in around 90% dead cells^9^.

We hypothesized that the higher level of localized cell death that was present with the TP would result in an increased level of inflammation throughout the tissue, which may be a stimulating factor for an enhanced immune response. This would be a natural response to systemic attack, and has been demonstrated on similar scales in the study of Leishmaniasis delivered by Sand Flies^23^. Figure 2d–f shows the results of the investigation we undertook to measure the cellular infiltration into the skin at 24 hrs post NP or TP application. In accordance with increased levels of cell death, we measured significantly higher numbers of infiltrating neutrophils and macrophages after TP than NP. There was also a trend towards a higher number of monocytes and eosinophils too (although statistically non-significant). This inflamed microenvironment may perform a similar role in the skin as traditional adjuvants, prompting dendritic cells to uptake vaccine and subsequently enhanced immunogenicity–a “physical adjuvant”^11^,^24^,^25^.

Following from the indications of higher cellular infiltration, we hypothesized an increase in immunogenicity would be observed with the TP. Correspondingly, Fig. 3 shows that for matched delivered doses of commercial influenza vaccine (Fluvax 2014) into skin (TP, NP, Intradermal injection) or muscle (Intramuscular injection), the TP surpassed all other delivery routes in immunogenicity. The TP immune response to 5 ng of vaccine is equivalent to the NP vaccine response to 50 ng of vaccine, which indicates a 10-fold decrease in the required vaccine dose to give comparable immunogenicity performance just by tailoring the geometry and subsequently, depth of delivery. Comparing similar doses (i.e., 5 ng TP vs 5 ng NP) the immune response from the TP is 2–3 times greater than that of the NP. Overall the immune data reflected that in our previous publications where NP > ID > IM, with similar dose responses to Fernando *et al.*^6^ but with the addition of the higher TP group. This matches our quantifications of relative cell death amounts, and those of previously published studies with ID/NP^11^. The increase in immunogenicity exhibited by TP application is similar to inclusion of a chemical adjuvant in the NP formulation, which was also demonstrated to increase immunogenicity^26^. However, this does not differentiate whether the improved immunogenicity stems from greater cellular damage or a greater depth of dose delivered. Using a different approach–a laser to induce tissue damage during vaccination–Wang *et al.*^24^ demonstrated increased immunogenicity, proposing this as a “sterile adjuvant” with energy of the same order of magnitude as other skin vaccination devices, at 35 mJ. The same group also proposed that a damage mechanism is what mosquitos use to ensure uptake of the malaria parasite–demonstrating increasing blood vessel damage at energies of 0.38–7.69 J 12. We suggest that the immunogenicity that can be generated following vaccination in skin is dependent upon the level of localized skin damage that is created and that by tuning this, a systemic immune response can surpass those achieved by other vaccination methods. The methods that achieve this, and have been discussed herein (the gene gun and laser adjuvant), use a surprisingly consistent energy of application in the range of 30–90 mJ, over an area of 16–70 mm^2^.

Within the field of vaccination, the NP had previously been observed to generate potent immune responses (in mice). One embodiment of this was equivalent immune responses to IM delivery, but with unprecedented antigen dose sparing^6^.

In this study, we have shown with the geometrical changes of the TP, that the same energy of patch application has been deployed more effectively to: (a) achieve a significantly deeper penetration (by 50%; thereby accessing most of the mouse dermis); (b) achieve a significant increase in vaccine delivery efficiency; (c) invoke distinct local immune responses (e.g. increased localized cell death; greater levels of subsequent cellular infiltrate); (d) produce greater systemic immune responses (than the Nanopatch), which we suggest is a result of this local cellular death environment. Further, by still using simple mechanical means of applying the TP against the skin (compared to, say, electrical energy), this approach is conceptually practical for broad utility in human vaccines, although with materials and sizes selected for optimal biocompatibility and immunogenicity. Upon translation to human use, these approaches will require similar assessment of inflammation together with the regular patient acceptability parameters (e.g., sensation, pain).

## Methods

### Manufacture of Patches

To manufacture patches we used our previously published dry etching method^17^. This allows high aspect ratio shapes to be manufactured with high yield on silicon wafers.

### Coating of Patches

NPs and TPs were coated in a coating solution of 1% Methylcellulose and the required vaccine dose, in injectable Phosphate Buffered Saline solution as per Chen *et al.*^18^. Fluvax 2014 was used as the antigen in this work (bioCSL, Victoria, Australia). Delivered dose was measured using a radio-assay described previously^6^. Once percentage of applied dose was known, this was used to define the amount of dose coating required for each patch design.

### Mice used

Specific pathogen free C57BL/6 female mice 6 to 8 weeks old were used in the study. Mice were house at the AIBN, University of Queensland animal facility. Groups of 5 were used in the experiments. Experiments were conducted under approval from the University of Queensland animal ethics committee, certificate number 556-12. Experiments were carried out in accordance with this approval.

### Application of patches to skin

This was performed as described by Crichton *et al.*, at a velocity of 2.3 m/s, to avoid variations from hand application^8^.

### Calculation of projections/protrusion surface areas

Solid Edge (Siemens PLM Software, Texas, USA) was used to create models from which surface area and volume could be measured, for given penetration into skin.

### Imaging puncture and delivery into skin

Nanopatches coated in Fluospheres (Molecular Probes, OR, USA) were applied to either live skin for histology or excised skin for CryoSEM and imaged as described previously^8^.

### Trans-Epidermal Water Loss

Measurement was made using a Tewameter TM300 manufactured by Courage + Khazaka Electronig GmbH (Cologne, Germany). Mice had TEWL measurements taken prior to patch application, immediately post patch application and at 30, 60, 90, 120 mins and then every hour from 3–8 hours after application. Two unpatched mice were also measured during these times to observe any environmental baseline changes in the animals.

### Imaging and viability staining of tissue

Viability staining of tissue and quantification of live/dead cells was performed as previously described^11^. A total of n = 4–5 fields of view (FOV) were acquired per sample, with n = 4 (NP) or 5 (TP) replicates per group.

### Analyzing skin-infiltrating cells by flow cytometry

Excised ear tissue was chopped and incubated with 1mg/mL Collagenase IV (Life Technologies, Carlsbad, CA) and 4U DNAse I (Thermo Fischer Scientific, Pittburgh, PA) for 30 minutes at (37 °C) before inactivating with 200 μL foetal bovine serum. Lysates were passed through a 70 μm strainer and cells pelleted by centrifugation with an additional 4U DNAse I Cells were incubated with purified anti-CD16/32 (Clone 93, Biolegend, San Diego, CA) for 15 minutes at 4 °C before washing and staining with a cocktail of fluorescently-conjugated anti-mouse monoclonal antibodies for 30 minutes at 4 °C: CD45.2 PercP Cy5.5 (Clone 104), CD11c PECy7 (N418), Ly6C APC (HK1.4RUO), (all from Affymetrix, San Diego, CA), CD11b Brilliant Violet 605 (M1/70), F4/80 Brilliant Violet 421 (BM8) (Biolegend, San Diego, CA) and Ly6G FITC (1A8), Siglec F PE (E50-2440) (Becton Dickinson, Franklin Lakes, NJ Data were acquired on a BD LSR II flow cytometer and analysed using Flowjo v9 (TreeStar, Ashland, OR)). DRAQ 7 (Biostatus, Shepshed, UK) was used to exclude dead cells before analysis. Doublets and debris were removed based on forward and side scatter properties before gating.

### Statistics

Results were compared using one-way ANOVA with Tukey post-test in Graphpad Prism Version 6.00.

### Measurement of immune response

IgG titers were determined using as described by Fernando *et al.*^27^ with the following changes: 5 μl of K- Blue TMB substrate (ELISA systems, Qld, Australia) was added and the color reaction was developed for 5 minutes in the dark. The reaction was stopped by the addition of 50 μl of 1 M phosphoric acid and the plates were read spectrophotometrically at 450 nm.

## Additional Information

**How to cite this article**: Crichton, M. L. *et al.* The changing shape of vaccination: improving immune responses through geometrical variations of a microdevice for immunization. *Sci. Rep.* **6**, 27217; doi: 10.1038/srep27217 (2016).

## Supplementary Material

Supplementary Information

## Figures and Tables

**Figure 1:**
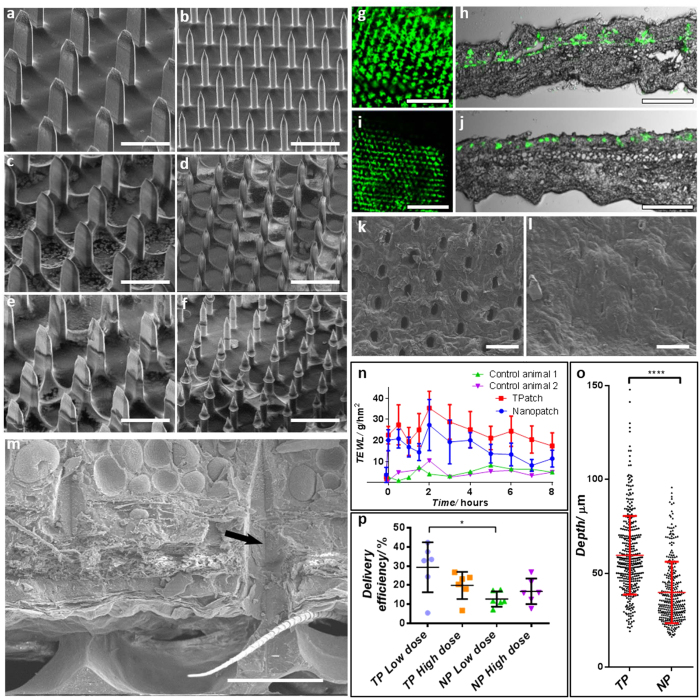
Uncoated, coated and post-application SEM images of TPs (**a**,**c**,**e** respectively) and NPs (**b**,**d**,**f** respectively); delivered fluorescent dye in skin, viewed form above and in transverse section for TPs (**g**,**h** respectively) and NPs (**i**,**j** respectively); top-down CryoSEM view of skin surface puncture during TP *in-situ* placement (**k**) and immediately following application showing closed sin perforations (**l**); (**m**) shows *in-situ* CryoSEM image of skin impressions from TPs, with an arrow indicating lines of the cutting/puncturing surface; a comparison of skin surface healing following TP and NP application is shown by TEWL changes over time (**n**); (**o**) shows that the TP reaches a significantly deeper location of antigen delivery in the skin, derived from fluorescent section measurement (***p < 0.001); delivered dose of vaccine is compared between high (50 ng) and low (5 ng) dose on the TP and NP, applied to skin in p (*p < 0.05). Statistics show mean ± standard deviation, significance is by one-way ANOVA. Bars **a–f, k, l** = 100 μm, **g, j** = 200 μm, **h, j** = 500 μm, m = 50 μm.

**Figure 2:**
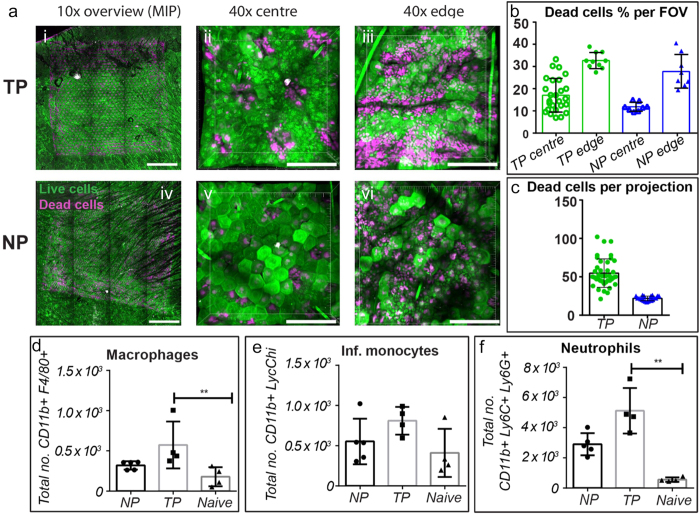
(**a**)–skin stained to show live (green) and dead (magenta) cells following TP and NP applications, at low magnifications and high magnifications of the center of the patches and at the edge; quantified levels of live/dead cells following TP and NP applications (**b,c**). (**d–f**) show changes in macrophage, infiltrating monocytes and neutrophils in skin following patch applications (**p < 0.01 using one-way ANOVA).

**Figure 3:**
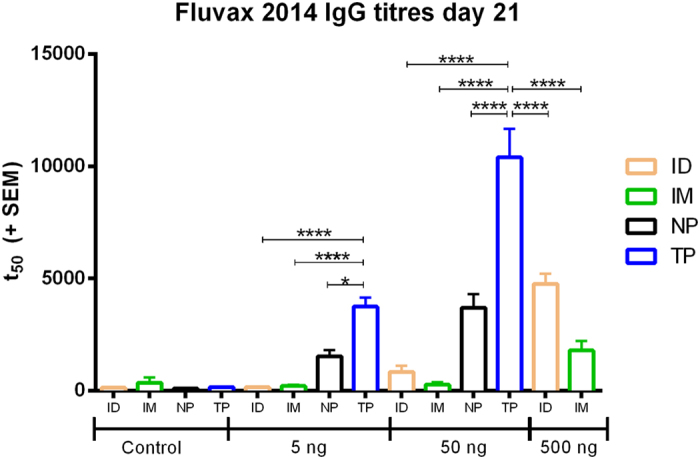
Day 21 anti-influenza antibody levels of sera from vaccinated C57BL/6 mice determined by indirect ELISA plotted as 50% titers. (*p < 0.01, ****p < 0.0001).
