# Corrigendum: The changing shape of vaccination: improving immune responses through geometrical variations of a microdevice for immunization

**DOI:** 10.1038/srep28722

**Published:** 2016-07-04

**Authors:** Michael Lawrence Crichton, David Alexander Muller, Alexandra Christina Isabelle Depelsenaire, Frances Elizabeth Pearson, Jonathan Wei, Jacob Coffey, Jin Zhang, Germain J. P. Fernando, Mark Anthony Fernance Kendall

Scientific Reports 6: Article number: 2721710.1038/srep27217; published online: 06022016; updated: 07042016

The original version of this Article contained typographical errors in the spelling of the author Alexandra Christina Isabelle Depelsenaire which was incorrectly given as Alexandra Christina Isobel Depelsenaire. This has now been corrected in the PDF and HTML versions of the Article.

