# Supplementary material for: The changing shape of vaccination: improving immune responses through geometrical variations of a microdevice for immunization
Source: Sci Rep. 2016 Jun 2;6:27217. doi: 10.1038/srep27217 (PMC4890175; doi:10.1038/srep27217)
Supplement: Supplementary Information [file srep27217-s1.pdf]

**Supplementary information**

**Title** The changing shape of vaccination: improving immune responses through geometrical variations of a microdevice for immunization

*Michael Lawrence Crichton*, David Alexander Muller[*], Alexandra Christina Isobel Depelsenaire, Frances Elizabeth Pearson, Jonathan Wei, Jacob Coffey, Jin Zhang, Germain JP Fernando, Mark Anthony Fernance Kendall[†]*

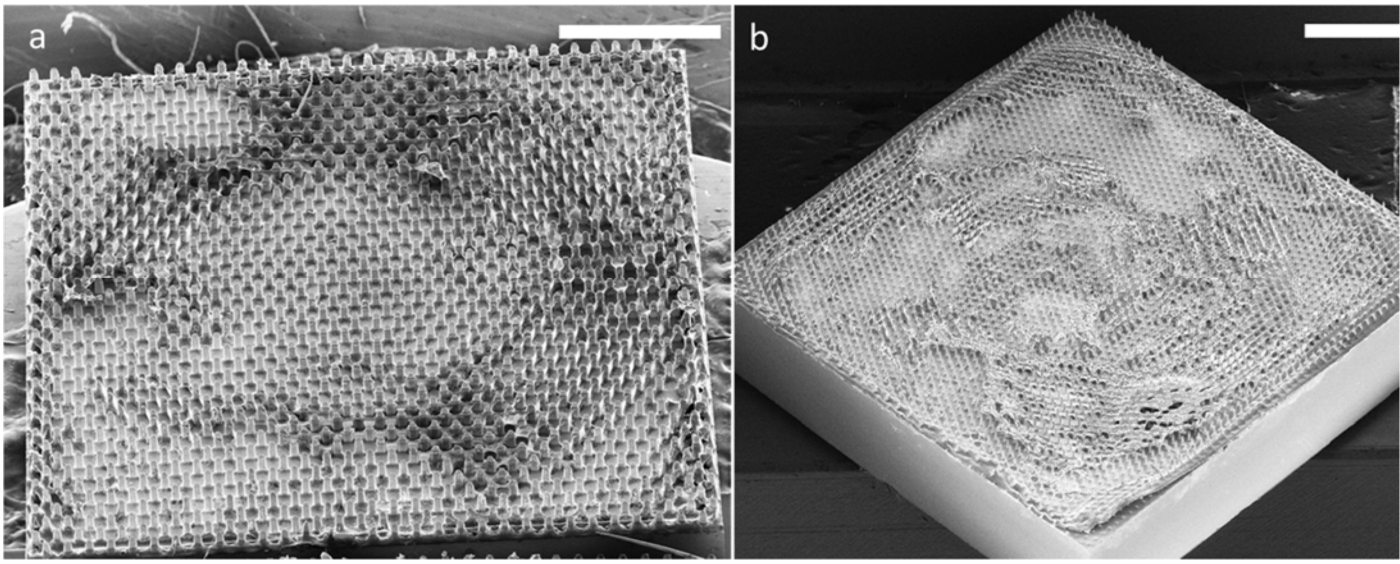


Figure S1 Caption: SEM image of TP and NP (a,b respectively) at low magnification showing the variation in vaccine coating release over the surface of the patch  Bright areas represent areas where coating has removed more completely whilst darker areas are still partially vaccine coated. Bar = 100 µm

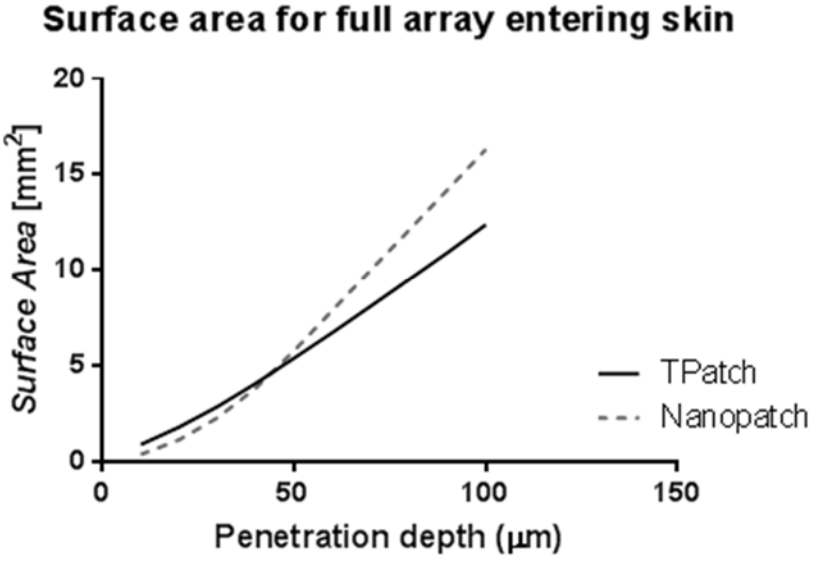


Figure S2 – Surface area of projections/protrusions entering skin for either the TP or NP, showing the similarities at the depths that patches reach in this study.
